# Consumption substitution and change of household indirect energy consumption in China between 1997 and 2012

**DOI:** 10.1371/journal.pone.0221664

**Published:** 2020-08-28

**Authors:** Zhipeng Tang, Shuang Wu, Jialing Zou

**Affiliations:** 1 Key Laboratory of Regional Sustainable Development Modeling, Institute of Geographic Sciences and Natural Resources Research, CAS, Beijing, China; 2 College of Resources and Environment, University of Chinese Academy of Sciences, Beijing, China; 3 Department of Geography, Hong Kong Baptist University, Hong Kong, China; 4 Guangdong Institute for International Strategies, Guangdong University of Foreign Studies, Guangzhou, China; The Bucharest University of Economic Studies, ROMANIA

## Abstract

With the rapid growth in the Chinese economy in recent decades, household incomes as well as consumption of goods and services have also steadily increased. This has resulted in growing demand for energy consumption across the economy. It has been suggested that consumption upgrades in tandem with substitutions might exert an impact on mitigating this growth. The input-output method was applied in this study to analyze variations in household indirect energy consumption between 1997 and 2012. The impact of consumption substitution on change was also determined using a two-tier structural decomposition analysis, in which the second-tier is a further decomposition based on first-tier results. The results show that the indirect energy use caused by household consumption makes up between 75% and 78% of total household energy demand and that this increased 161.2% over the study period. First-tier decomposition results reveal that this change was mostly caused by household consumption scale and energy intensity effects. Second-tier decomposition results reveal strong evidence for consumption substitution between energy-intensive industries and non-energy-intensive ones and that this can have an impact on reducing household indirect consumption. Household consumption therefore plays a prominent role in total energy consumption. Transforming to non-energy-intensive or services led consumption patterns should therefore be encouraged by the Chinese government in order to achieve conservation goals.

## 1. Introduction

Global warming has been a key research topic for several decades. This phenomenon has mainly been attributed to rapidly intensifying greenhouse gas emissions, caused largely by increasing energy consumption. China has experienced substantial economic growth and rapid urbanization over this time period [1], mainly driven by massive energy consumption, almost exclusively fossil fuels. According to BP [2], China became the country with the highest energy consumption in 2009 and remains so today, responsible for about 23.2% of global energy consumption in 2017, about 449 million tons of coal equivalent (Mtce). China is now focused on environmental issues, undertaking in 2009 to cut its carbon intensity by 40%-45% by 2020 compared with 2005 levels. At the Paris Climate Change Conference, China committed to peak total carbon emissions by the 2030s, showing great ambition to control both energy consumption and carbon emissions. This goal, to maintain energy conservation and a continuing reduction in CO_2_ emissions, was documented in the Chinese ‘Thirteenth Five-Year Plan’ (2016–2020).

Society has paid increased attention to energy consumption linked to production for some time now instead of focusing on households. Although recent statistical data show that household energy consumption has been significant in China, this information refers only to household direct consumption. It is clear that household indirect energy consumption is also important, and most of this energy is consumed indirectly by the goods used by households. Thus, indirect energy consumption and resultant CO_2_ emissions are much higher than those originating from direct sources [3]. There has been a surge of recent research interest in both direct and indirect household energy consumption [4, 5] concerned with calculating the latter of these variables as well as underlying forces. The Chinese government advocates vigorously upgrading consumption in order to stimulate industrial upgrade. Thus, as a result of this process, positive impacts might also occur in terms of mitigating environmental pressure. This study investigates the influences of consumption substitution on Chinese household indirect energy consumption and further quantifies the effect of within, and between, sectoral consumption substitution, then some policy implications were proposed to reduce Chinese household indirect energy consumption.

## 2. Literature review

### 2.1. The factor decomposition method

Two common decomposition methods are commonly applied, index decomposition analysis (IDA) and structural decomposition analysis (SDA). These approaches have been extensively applied in order to investigate the mechanisms that influence energy consumption, carbon emissions, and other environmental variables [6–8]. Indeed, IDA is often employed to analyze the mechanisms underlying environmental issues within specific industries because this approach has clear advantages including being concise and having low data requirements. The drawbacks of IDA include the fact that it does not produce detailed decompositions for consumption structure. As this approach does not rely on input-output tables, it is also limited when analyzing interdependencies between different industries. It is therefore impossible to use IDA to quantify the indirect effects of change in final consumption because this method cannot distinguish between intermediate and final demand [9]. The most commonly applied, preferable IDA method is the logarithmic mean Divisia index (LMDI) [10], first proposed by Ang and Choi [11]. This approach is popular for analyzing energy consumption and environmental issues in economic systems [12–15]. By applying the LMDI method, Fu et al. [16] found that the economic output effect is the key determining factor contributing to increases in carbon emissions and that a structural effect also had a detrimental effect on the Chinese petrochemical industry between 2000 and 2010. Other scholars in the energy-intensive industries have produced similar results. Lin et al. [17] showed that the industrial scale effect further increases CO_2_ emissions while the energy intensity effect is the main variable driving a decrease in these emissions from energy-intensive industries. Similarly, Wang et al. [18] decomposed the carbon intensity change of energy-intensive industries by implying LMDI; these studies showed that different factors have exerted variables effects on industries and therefore suggested that policies should vary according to sector. It is noteworthy that the LMDI method also cannot take industrial linkages into account.

In contrast, SDA is another widely used decomposition method that has been applied to examine multi-industry environmental issues [19]. This approach has the advantage that it takes industrial interdependencies into account so distinctions can be made between intermediate and final demand. This approach has therefore frequently been applied to assess the factors influencing energy consumption and CO_2_ emissions. In earlier studies, Weber [20] used SDA to measure the structural effects contributing to the energy consumption in the United States between 1997 and 2002, while José et al. [21] used this approach to determine the factors driving CO_2_ emissions within the Spanish economy. A number of other studies have focused on the factors underlying environmental issues in China; Mi et al. [22] used SDA to examine the driving factors of carbon emissions in China from 2005 to 2012, and Chang et al. [23] identified the significant effects and sectors that influenced CO_2_ emissions between 2005 and 2010. Yuan and Zhao [24] also used a sub-system input-output decomposition method to analyze changes in energy-intensive industry carbon emissions; these works showed that an external component is the key determining factor contributing to growth in CO_2_ emissions. In another study, a modified SDA method was used to investigate the relationship between structural effects and emissions from the power generation industry [25].

With its ongoing development, SDA is becoming ever more powerful for analyzing the mechanisms involved in environmental issues. One important progress is the two-tiered KLEM (capital, labor, energy, material) decomposition model [26], where input technology coefficients are furtherly decomposed. Another crucial development in SDA is the adoption of ideal decomposition methods [27]. In order to overcome the shortcomings of the KLEM model, the D&L [28] and LMDI [29] methods have been applied as components of SDA for ideal decomposition. These developments have been applied in recent work; Zhou et al. [30] used a three-tier SDA approach to analyze the main driving factors that affect the change of energy intensity in China with stress on information and communication technology and production structure. Yu et al. [31] used a two-tiered attribution SDA method to reveal the drivers of energy consumption within the Beijing-Tianjin-Hebei region.

### 2.2. Household indirect energy consumption

Household energy consumption comprises direct and indirect components. As no statistics are available for the indirect component, a calculation is necessary. Two common methods are used to estimate indirect household energy consumption, including process analysis which sums all the energy input into each stage of the production process. Although possible, this method is hugely time consuming and expensive [32]. The second approach is the input-output method which makes estimates using Leontief inverse intermediate energy inputs for one or more industries [33–36].

In addition to research aimed at calculating household indirect energy consumption, additional works have focused on regional household energy requirements in countries and regions including India [37], Brazil [5], and the European Union [38]. As is also the case in China [3, 39], results show that the amount of energy consumed by indirect household consumption is actually much greater than that consumed directly. The CO_2_ emission due to by household indirect energy consumption is also important; several research teams have addressed this issue [40–43] and have shown that these emissions account for between 77% and 84% of total household CO_2_ output.

Decomposition methods are frequently used [44] for analyzing the factors that drive changes in household indirect energy consumption. These methods have helped to show that an increasing trend in household indirect energy is mainly the result of consumption scale, while decreases in energy intensity have also contributed significantly to reductions in household indirect energy consumption. On this basis, Yuan et al. [44] advocated that energy-intensive sectors with relatively higher direct or total energy consumption are key areas where considerable improvements could be made in China to reduce energy consumption and CO_2_ emissions.

The relationship between consumption substitution and energy use has rarely been studied. Earlier research has mainly focused on energy substitutions [44–46], specifically how cleaner sources could substitute for coal and other fossil fuels. Previous research has contributed to calculations and our understanding of trends, structures, and reductions in Chinese household indirect energy consumption. At the same time, however, consumption substitution is another crucial analytical factor which has been neglected due to a lack of detailed data or appropriate techniques. Due to the considerable differences in energy intensity of energy-intensive sectors and non-energy-intensive sectors, so in this research the sectors are classified into energy-intensive sectors group and non-energy-intensive sectors group. Concerning the consumption substitution effect in analyses will enable a deeper understanding of changes in energy consumption change across China. The aim of this research is therefore to calculate household indirect energy consumption by sector in China between 1997 and 2012 as well as within sub-periods. The goal of this research is to shed light on the relationship between consumption substitution and household indirect energy consumption. A number of practical suggestions are proposed to reduce Chinese energy consumption.

## 3. Data and methods

### 3.1. Data sources

Calculating Chinese household indirect energy consumption required analysis of 1997, 2002, 2007, and 2012 input-output tables. These tables have different sector aggregations, however; 124 for 1997, 122 for 2002, 135 for 2007, and 139 for 2012. Thus, in order to remain consistent with respect to energy consumption sectors in ‘China’s Statistical Year-book 2008–2013’ [47], original sectors were merged into 27 for input-output tables (Table 1) and prices were adjusted to 1997 values. All energy consumption data came from ‘China’s statistical year-book 2008–2013’ and sectors were divided into two groups based on energy-consumption level using the *Statistical Bulletin of the People’s Republic of China on National Economic and Social Development in 2010* [48]. Two groups were defined, ‘energy-intensive’ and ‘non-energy-intensive.’ The first of these contains S11 (the processing of petroleum, coking, and processing of nuclear fuel), S12 (the manufacture of chemicals and chemical products), S13 (the manufacture of non-metallic mineral products), S14 (the smelting and pressing of ferrous metals), S15 (the smelting and pressing of non-ferrous metals), and S22 (the production and supply of electric power and heat power). And the non-energy-intensive sector groups are summarized in Table 1; energy-intensive ones are labeled *EI*, while others are denoted *NEI*.

**Table 1:** Sector code and description within the Chinese economy.

Sector code	Sector description	Sector type
S1	Agriculture	*NEI*
S2	Ming and washing of coal	*NEI*
S3	Extraction of petroleum and natural gas	*NEI*
S4	Mining and processing of metal ores	*NEI*
S5	Mining and processing of non-metal ores	*NEI*
S6	Manufacture of foods, beverages, and tobacco	*NEI*
S7	Manufacture of textile	*NEI*
S8	Manufacture of textile wearing apparel, footwear, caps, leather, fur, feather, and related products	*NEI*
S9	Processing of wood and manufacture of furniture	*NEI*
S10	Manufacture of paper, printing, and products for culture, education, and sports	*NEI*
S11	Processing of petroleum, coking, processing of nuclear fuel	*EI*
S12	Manufacture of chemicals and chemical products	*EI*
S13	Manufacture of non-metallic mineral products	*EI*
S14	Smelting and pressing of ferrous metals	*EI*
S15	Smelting and pressing of non-ferrous metals	*EI*
S16	Manufacture of metal products	*NEI*
S17	Manufacture of general and special-purpose machinery	*NEI*
S18	Manufacture of transport equipment	*NEI*
S19	Manufacture of electrical machinery and equipment	*NEI*
S20	Manufacture of communication equipment, computers, and other electronic equipment	*NEI*
S21	Other manufacturing	*NEI*
S22	Production and supply of electric power and heat power	*EI*
S23	Production and supply of gas	*NEI*
S24	Production and supply of water	*NEI*
S25	Construction	*NEI*
S26	Transport, storage, and post	*NEI*
S27	Other services	*NEI*

Source: referenced from Zou J, Liu W, Tang Z [49].

### 3.2. Household indirect energy consumption

In order to develop an in-depth analysis of energy consumption within industrial chains it is necessary to initially consider the input of intermediate production processes by industries with their energy use at the same time in the industrial chains [50]. An environmental input-output model (EIO) is an appropriate solution to calculate energy consumption, including direct and indirect consumption within an economic system. The EIO method provides an extended analysis based on a standard Leontief input-output model [51, 52]. This approach quantifies indirect energy use from the perspective of household consumption; all monetized commodity consumed directly by households can be converted into energy consumption, denoted as indirect household energy consumption. This aspect of the EIO model is calculated as follows:

$$F=e{\left(I–A\right)}^{-1}Y=e\times C\times Y.$$ (1)

In this expression, *I* denotes the identity matrix which are ones on diagonal, while *A* is the intermediate use matrix, *e* indicates energy per Yuan output consumed, and *C* is the Leontief inverse, (*I*−*A*)^−1^. Similarly, *Y* is the column vector of final demand and *F* represents embodied energy consumption caused by final demand. Thus, incorporating household consumption, *s*, into Eq (1), household indirect energy consumption can be calculated as follows:

$$E=e(I–A{)}^{-1}s.$$ (2)

In this expression, *E* denotes the total embodied energy use caused by household consumption. Column vector *s* can be expressed by matrix *z* multiplied by *q*, while *z* represents a matrix of household consumption industrial structure and *q* denotes a column vector of household consumption scale, as follows:

s=z×q. (3)

It is clear that Eq (2) can rewritten as follows:

$$E=e(I–A{)}^{-1}s=e\times C\times z\times q.$$ (4)

### 3.3. First-tier decomposition of household indirect energy consumption

It is necessary to initially calculate household indirect energy consumption by sector in order to decompose changes in this variable. The national EIO model was therefore applied to establish a decomposition method and to analyze the factors driving changes in household indirect energy consumption. Research by Ang [53] has shown that the LMDI approach is superior to other index composition methods because it is both accurate in decomposition and consistent in aggregation. Indeed, one practical guide for the LMDI method even includes a technique for handling zero values derived from the work of Ang [54] and Ang and Liu [55]. Hence we carried out decomposition analysis of household indirect energy consumption by combining the EIO model and the LMDI approach together. Thus, building on Eq (4), variation in *E* over the period between *t*_0_ and *t*_1_ can be described as follows:

$$\Delta E=E^{t_{1}}–E^{t_{0}}=\Delta e^{T}+\Delta C+\Delta z+\Delta q.$$ (5)

In this expression, *Δ* denotes variation in each variable while superscript *T* is the transpose of this vector. Similarly, *s*_*j*_ is the column vector of consumption structure; this vector can also be expressed via the product of matrix *z*_*jm*_ multiplied by column vector *q*_*m*_, as follows:

$$s_{j}=\sum_{m}s_{jm}=\sum_{m}z_{jm}q_{m}.$$ (6)

Applying the LMDI approach, Eq (4) can be rewritten as follows:

$$E=\sum_{j}\sum_{i}\sum_{m}(e_{i}C_{ij})z_{jm}q_{m}.$$ (7)

This means that according to Eq (7) and building on the earlier work of Ang [54] and Ang and Liu [55], variation in each term within Eq (5) is as follows:

$${\Delta e}^{T}=\sum_{j}L(E_{j})\sum_{i}\frac{L(g_{ij})}{L(g_{j})}\cdot \ln\left(\frac{e_{j}^{t_{1}}}{e_{j}^{t_{0}}}\right);$$ (8)

$$\Delta C=\sum_{j}L(E_{j})\sum_{i}\frac{L(g_{ij})}{L(g_{j})}\cdot \ln\left(\frac{C_{ij}^{t_{1}}}{C_{ij}^{t_{0}}}\right);$$ (9)

$$\Delta z=\sum_{j}L(E_{j})\sum_{m}\frac{L(s_{jm})}{L(s_{j})}\cdot \ln\left(\frac{z_{jm}^{t_{1}}}{z_{jm}^{t_{0}}}\right)$$ (10)

and;

$$\Delta q=\sum_{j}L(E_{j})\sum_{m}\frac{L(s_{jm})}{L(s_{j})}\cdot \ln\left(\frac{q_{m}^{t_{1}}}{q_{m}^{t_{0}}}\right).$$ (11)

Terms *Δ*^*eT*^, *ΔC*, *Δz*, and *Δq* in these expressions denote changes in the four effects resulting from energy intensity, input and household consumption structures, and scale in indirect household energy consumption from *t*_0_ to *t*_1_, respectively. Thus, on the basis of Eqs (5)–(11), changes in Chinese household indirect energy consumption were obtained as a result of these four factors. Note that *L*(*x*) denotes the function of the logarithmic mean Divisia weight value; in the case of independent variables (denoted *x*), including the indirect final consumption energy of different sectors, *E*_*j*_, a corresponding logarithmic mean Divisia weight value *L*(*E*_*j*_) between *t*_0_ and *t*_1_ was obtained. A detailed explanation regarding the derivation of sub-effects in Eq (10) and Eq (11) is presented in S1A Appendix. Expressions are as follows:

$$L(E_{j})=\frac{E_{j}^{t_{1}}-E_{j}^{t_{0}}}{\ln\;E_{j}^{t_{1}}-\ln\;E_{j}^{t_{0}}};$$ (12)

$$L(g_{ij})=\frac{g_{ij}^{t_{1}}-g_{ij}^{t_{0}}}{\ln\;g_{ij}^{t_{1}}-\ln\;g_{ij}^{t_{0}}}$$ (13)

$$L(g_{j})=\frac{g_{j}^{t_{1}}-g_{j}^{t_{0}}}{{lng}_{j}^{t_{1}}-lng_{j}^{t_{0}}}.$$ (14)

Therefore:

$$g_{ij}=e_{i}C_{ij}$$ (15)

$$g_{j}=\sum_{i}g_{ij}.$$ (16)

### 3.4. Second-tier decomposition of household consumption structure

Enlightened from Rose and Chen’s work [26], it is clear that changes in the structure of household consumption can be related to increases in consumption capacity leading to proportional changes in products (e.g., a decrease in Engel’s coefficient leads to a food consumption proportional decrease). At the same time, changes in structure can also be related to the substitution of different consumption products due to consumer differences.

Consumption products can be categorized as either energy-intensive or non-energy-intensive while household consumption structure, *z*_*jm*_, can be divided into energy-intensive, $z_{jm}^{EI}$, and non-energy-intensive products, $z_{jm}^{NEI}$. In this analysis, $z_{jm}^{EI}$ denotes the matrix of energy-intensive consumption industrial structure containing only all the rows of sector j of energy-intensive consumption, with zeros elsewhere; similarly, $z_{jm}^{NEI}$ denotes the matrix of non-energy-intensive consumption industrial structure. This expression satisfies Eq (17), as follows:

$$z_{jm}=z_{jm}^{EI}+z_{jm}^{NEI}.$$ (17)

In order to develop an in-deep analysis of the sources of change underlying embodied energy consumption in intensive products, a splitting analysis was performed within second-tier decomposition following Casler and Rose [56]. Differences in industrial household consumption structure can therefore be divided into three mutually exclusive parts, as follows:

$$\Delta z=\Delta z_{in}+\Delta z_{be}+\Delta z_{co}.$$ (18)

The first term in this expression, $\Delta z_{in}=r_{t_{0}\to t_{1}}^{EI}z_{jm(t_{1})}^{EI}+r_{t_{0}\to t_{1}}^{NEI}z_{jm(t_{1})}^{NEI}-z_{jm(t_{0})}^{EI}-z_{jm(t_{0})}^{NEI},$ measures the effects of consumption substitution within energy-intensive sectors so that the group of non-energy-intensive sectors aggregate between *t*_0_ and *t*_1_. Similarly, the second term, $\Delta z_{be}=r_{t_{0}\to t_{1}}^{EI}z_{jm(t_{1})}^{NEI}+r_{t_{0}\to t_{1}}^{NEI}z_{jm(t_{1})}^{EI}-r_{t_{0}\to t_{1}}^{EI}z_{jm(t_{1})}^{EI}-r_{t_{0}\to t_{1}}^{NEI},$ measures the effect of consumption substitution between energy-intensive sectors and the non-energy-intensive group between *t*_0_ and *t*_1_. The third term, $\Delta z_{co}=z_{jm(t_{1})}^{EI}+z_{jm(t_{1})}^{NEI}-r_{t_{0}\to t_{1}}^{EI}z_{jm(t_{1})}^{NEI}-r_{t_{0}\to t_{1}}^{NEI}z_{jm(t_{1})}^{EI}$, therefore measures the effect of household consumption level change between *t*_0_ and *t*_1_. The ratio can therefore be expressed as follows:

$$r_{t_{0}\to t_{1}}^{EI}=\frac{\sum_{j}z_{jm(t_{1})}^{EI}}{\sum_{j}z_{jm(t_{0})}^{EI}}.$$ (19)

This expression controls the change in unit energy-intensive industry consumption costs between *t*_0_ and *t*_1_. Similarly, the following ratio controls the change in unit non-energy-intensive consumption costs between *t*_0_ and *t*_1_:

$$r_{t_{0}\to t_{1}}^{NEI}=\frac{\sum_{j}z_{jm(t_{1})}^{NEI}}{\sum_{j}z_{jm(t_{0})}^{NEI}}.$$ (20)

## 4. Results

### 4.1. Chinese household energy consumption

#### 4.1.1. Sectoral energy intensity

**Fig 1:**
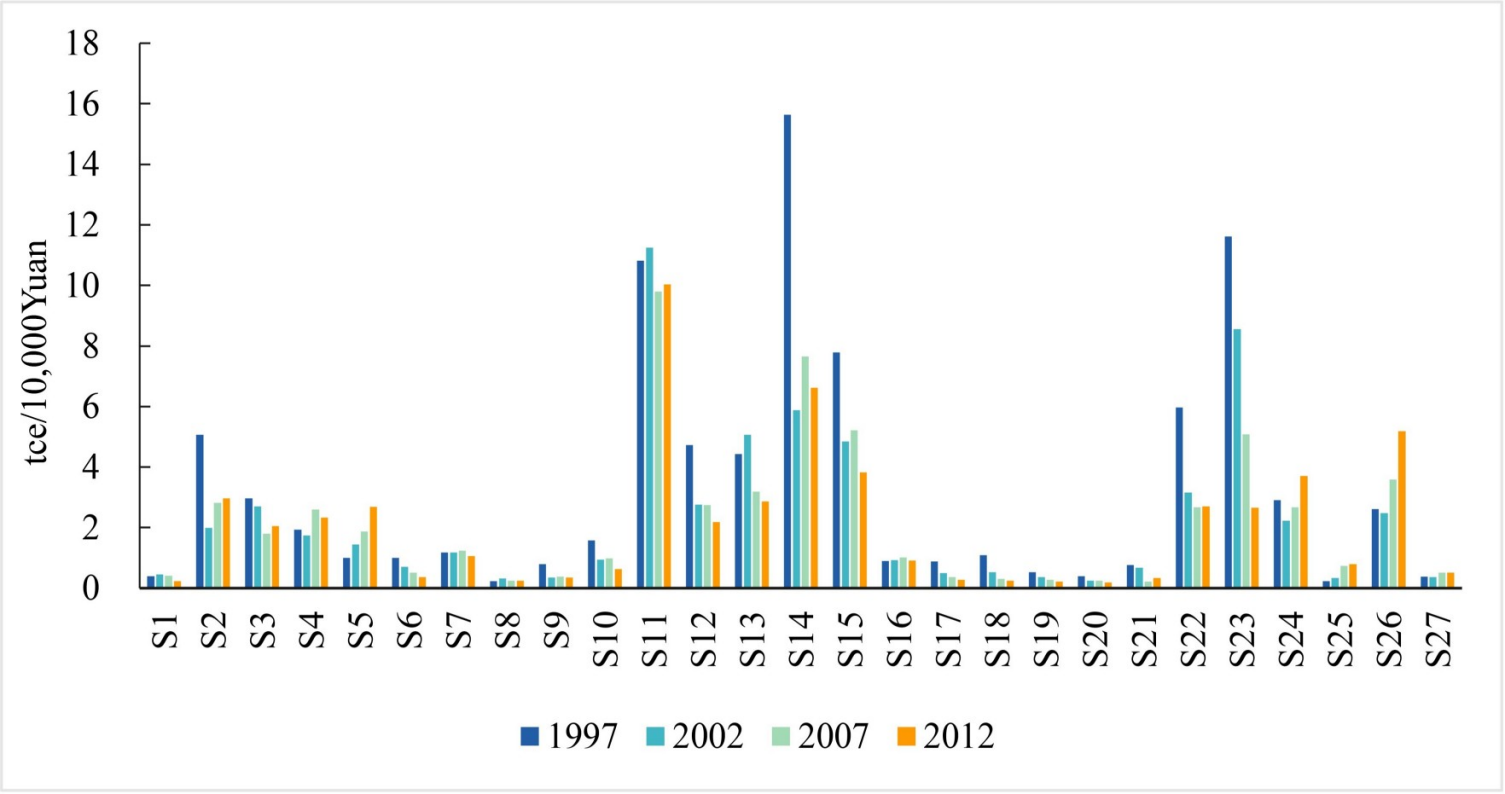
Comparison of Chinese sectoral energy intensities in 1997, 2002, 2007, and 2012.4.1.2. Household indirect energy consumption.

A detailed analysis was performed to verify changes in the energy efficiency of specific sectors. The data presented in Fig 1 compare the energy intensities of 27 sectors in 1997, 2002, 2007, and 2012. Results show that the intensities of energy-intensive industries (i.e., S11–S15, S22) are much higher than those of sectors within the non-energy-intensive group. It is also clear that a significant divergence in sectoral energy intensity exists within energy-intensive and the non-energy-intensive groups; in one example, the intensity of the *production and supply of gas (S23)* within non-energy-intensive group is much higher than that in other sectors within this group. The majority of sectors have experienced significant improvements in energy efficiency. In contrast, a few sectors within energy-intensive industries maintained incremental improvements over the study period, including *construction (S25)* and *transport*, *storage and post (S26)*. Amongst energy-intensive industries, *smelting and pressing of ferrous metals (S14)* and *production and supply of electric power and heat power (S22)* decreased in energy intensity the most between 1997 and 2012, which decrease from 15.6tce/10^4^Yuan in 1997 to 6.6tce/10^4^Yuan and from 15.6tce/10^4^Yuan in 1997 to 2.7tce/10^4^Yuan, respectively. Results also show that actually not all sectoral energy intensities decreased for all sub-periods; values for the *pressing of ferrous metals (S14)* and the *smelting and pressing of non-ferrous metals (S15)* exhibited a reversed trend between 2002 and 2007.

**Fig 2:**
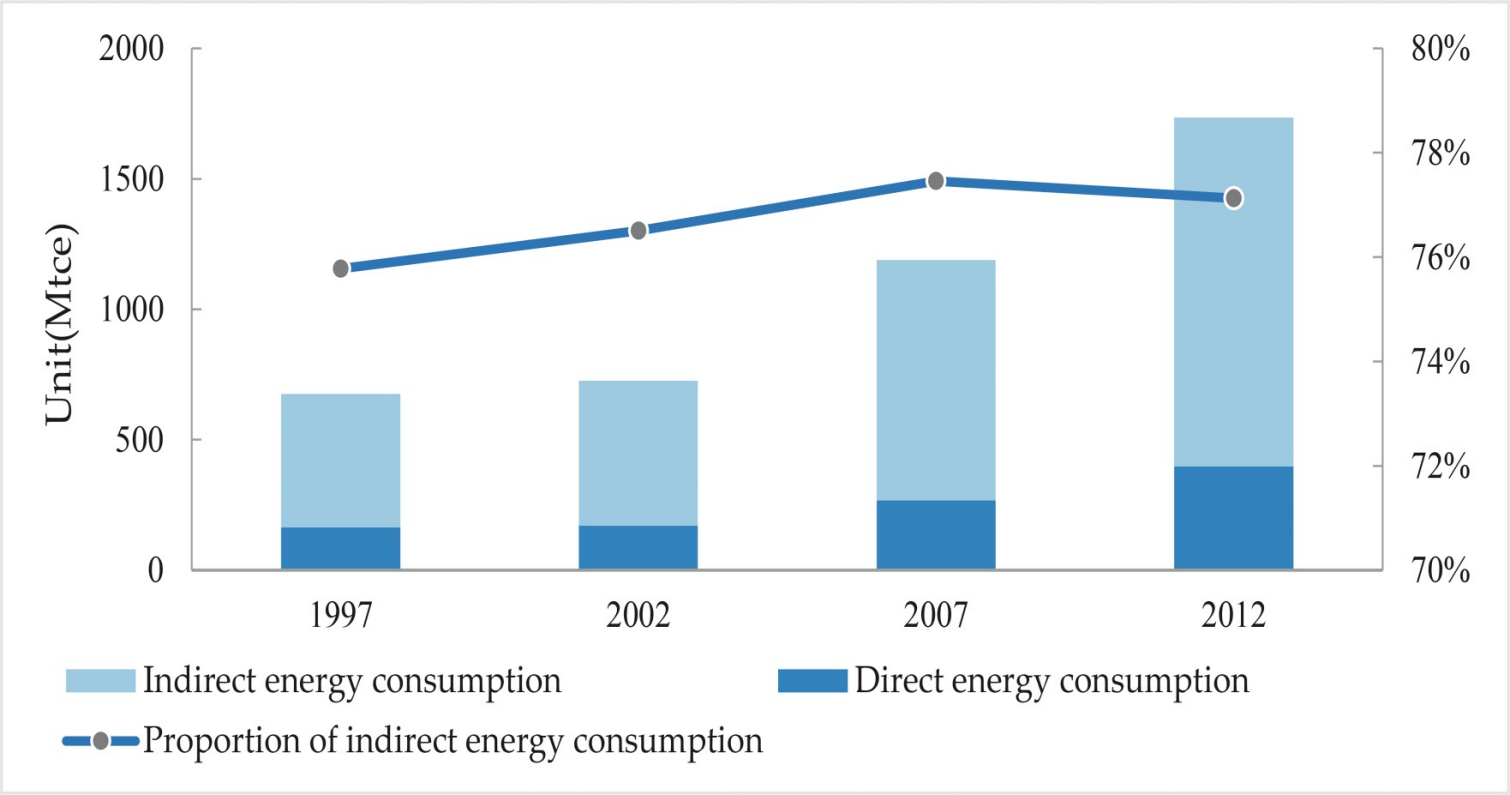
Direct and indirect energy consumption at the household level between 1997 and 2012.

Values for household indirect energy consumption across China were calculated for 1997, 2002, 2007, and 2012 on the basis of Eq (2). The results presented in Fig 2 show that total household indirect energy consumption increased between 1997 and 2012 (i.e., 512.1 Mtce in 1997 to 1,337.6 Mtce in 2012), an overall increase of 161.2%. Data show that household indirect energy consumption did not change a great deal between 1997 and 2002 while a 65.9% increase was seen between 2002 and 2007. This increasing trend then slowed slightly between 2007 and 2012. The proportion of indirect household energy consumption accounts for between 75% and 78% of the total throughout this whole period.

**Fig 3:**
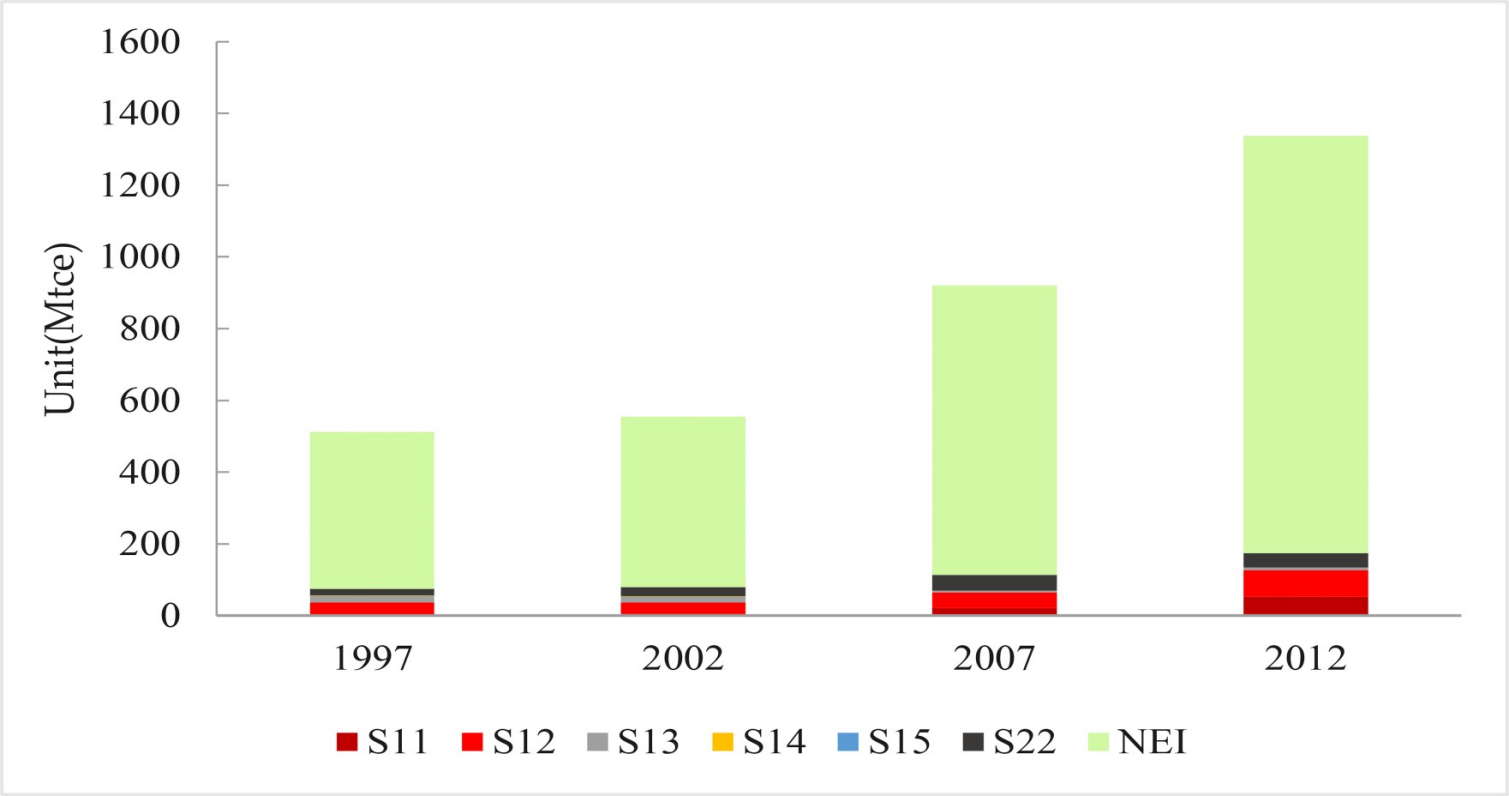
Chinese sectoral household indirect energy consumption in 1997, 2002, 2007, and 2012.

The data presented in Fig 3 show that the indirect energy consumption of energy-intensive industries accounts for between 12% and 15% of total indirect household energy consumption, much less than the proportion consumed by energy-intensive sectors. The proportion of indirect household energy consumption has also fallen slightly over time, from 14.4% in 1997 to 13.0% 2012. In contrast, indirect energy consumption caused by non-energy intensive sectors accounts for a large proportion of the total, mainly due to the large consumption proportion in non-energy intensive sectors.

### 4.2. First-tier decomposition results

In the first-tier decomposition, the household energy consumption change was decomposed into four factors, which were the energy intensity effect, intermediate input mix effect, household consumption mix effect, and household consumption scale effect. The results by sectors was aggregated into two groups: energy-intensive sectors and non-energy-intensive sectors (Table 2).

**Table 2:** The contribution of driving factors to household indirect energy consumption change at the sectoral level between 1997 and 2012 (Unit: %).

Period	Group	Energy intensity effect	Intermediate input structure effect	Household consumption structural effect	Household consumption scale effect	Total Change
**1997–2002**	Energy-intensive	-38.1	0.2	5.6	39.7	7.4
	Non-energy- intensive	-29.2	1.4	-0.3	36.6	8.5
**2002–2007**	Energy-intensive	-38.8	32.3	-1.0	50.4	42.9
	Non-energy- intensive	-9.9	23.7	1.2	54.7	69.8
**2007–2012**	Energy-intensive	-4.6	0.2	1.8	56.5	53.9
	Non-energy- intensive	-7.2	-4.6	2.1	53.7	44.1
**1997–2012**	Energy-intensive	-107.7	38.8	19.7	185.3	136.1
	Non-energy- intensive	-73.2	25.0	12.3	201.3	165.5

Results show that household indirect energy consumption in China has increased by 136.1% and 165.5% within the energy-intensive and non-energy-intensive groups, respectively, between 1997 and 2012. In terms of factors, data show that the energy intensity effect has been key in driving the decrease in household indirect energy consumption throughout all periods; this outcome shows that China has achieved significant improvements in energy efficiency over time. More specifically, it is clear that the energy intensity effects of intensive and non-intensive groups are very different; the energy intensity effect in the energy-intensive group was much stronger than that in the non-energy intensive group between 1997 and 2012. This means that the energy-intensive effect has been much more important in the first group than has been the case in the latter. In contrast, the household consumption scale effect is the most important factor in enhancing increases in indirect energy consumption; indirect energy consumption values increased by 185.3% and 201.3% in the two groups over the study time period, respectively. These results are consistent with rapid development of the Chinese economy.

It is also the case that intermediate input structural effects have varied markedly during different periods but have exerted limited impacts on indirect energy consumption over the timescale of this analysis. It is noteworthy that between 2002 and 2007, the energy structure transitioned towards coal [57]. Data summarized in Table 2 reveal that the household consumption structural effect has not been significant in driving indirect energy consumption changes compared to other factors; other variables are at play in explaining further decomposition of the consumption structural effect. In the first place, it is clear that consumption has played a more significant role in the development of the Chinese economy; consumption contributed 54.5% of gross domestic product in 1997, rose to 54.9% in 2012, and then surged to 76.2% in 2018. Second, consumption structure is changing rapidly and so its effects on energy consumption remain under consideration. In one example, if we consider food, clothes, and housing to be basic necessity consumption variables, it is clear that there has been an overall reduction in these commodities from 69.9% in 1997 to 58.1% in 2012. Research results released by the Department of Economic Forecasting of The State Information Center of China [58] suggest that the consumption of basic necessities will continue to decline in China and will exert a significant impact on energy consumption. This structural phenomenon may also be highly aggregated and will therefore dramatically underestimate the overall consumption substitution effect.

### 4.3. The consumption substitution effect

On the basis of the methods analyzed in Section 2, a two-tier decomposition method was used to further reduce the household consumption structural effect into three factors, within group substitution, between group substitution, and consumption level effects. This discussion will mainly focus on the impact of the first two consumption substitution effects on household indirect energy consumption. It is clear that one of these, the between-group consumption substitution effect, refers to how consumption substitutes across a sector and considers impact on indirect energy consumption. The second, the within group effect, considers consumption substitution within either energy-intensive or non-energy intensive sectors to evaluate how much this will impact household indirect energy consumption.

#### 4.3.1. The within-group consumption substitution effect

The second-tier decomposition results by the group in Table 3 show that the within-group effect in energy-intensive and non-energy-intensive sectors has contributed to an increase in indirect energy consumption over all sub-periods between 1997 and 2012. Indeed, throughout the entire period considered here, the within-group substitution effect has increased by 17.83 Mtce in energy-intensive sectors and by 52.79 Mtce in their non-energy-intensive counterparts.

**Table 3:** Attribution of consumption substitution effects to household indirect consumption changes between 1997 and 2012 (Unit: Mtce).

Period	Groups	Within-group substitution effect	Between-group substitution effect	Consumption level effect	Total household consumption structural effect
**1997–2002**	Energy-intensive	1.07	3.24	-0.22	4.10
	Non-energy-intensive	-0.06	-17.24	16.08	-1.22
**2002–2007**	Energy-intensive	3.62	-4.73	0.29	-0.81
	Non-energy-intensive	4.03	30.39	-28.54	5.89
**2007–2012**	Energy-intensive	4.44	-2.45	0.10	2.08
	Non-energy-intensive	16.22	24.23	-23.21	17.23
**1997–2012**	Energy-intensive	17.83	-3.43	0.13	14.53
	Non-energy-intensive	52.79	30.57	-29.33	54.04

The effects on sectoral household energy consumption are shown in Table 4. It can be seen that the consumption substitution effect increased household sectoral energy consumption in ‘*S11—processing of petroleum*, *coking*, *processing of nuclear fuel* (30.98 Mtce),’ ‘*S18—manufacture of transport equipment* (23.99 Mtce),’ ‘S26—*transport*, *storage and post* (26.15 Mtce),’ and in ‘*S27—other services (148*.*98 Mtce)*’ between 1997 and 2012. This means that the consumption of products in these sectors have substituted for others within energy-intensive and non-energy-intensive groups. At the same time, however, the within-group consumption substitution effect decreased indirect sectoral household energy consumption in ‘*S1—agriculture* (-74.27 Mtce),’ ‘*S8—manufacture of textile wearing apparel*, *footwear*, *caps*, *leather*, *fur*, *feather*, *and related products* (-17.45 Mtce),’ and ‘*S13—manufacture of non-metallic mineral products* (-21.64 Mtce).’ Similarly, these implicate that other sectoral consumptions substituted these sectoral consumptions within the same group.

**Table 4:** Comparison of within-group consumption substitution effects: Contributions to changes in household indirect energy consumption (unit: Mtce).

Sector code	1997–2002	2002–2007	2007–2012	1997–2012
**S1**	-22.37	-39.55	-6.89	-74.27
**S2**	3.10	-3.28	-1.57	-0.86
**S3**	0.50	-1.08	0.00	-0.29
**S4**	0.00	0.00	0.00	0.00
**S5**	-0.03	-0.42	0.00	-0.34
**S6**	-33.91	22.80	14.85	-11.98
**S7**	-1.22	-9.81	-0.30	-11.07
**S8**	-13.89	8.47	-8.18	-17.45
**S9**	-2.73	-1.84	0.54	-5.56
**S10**	-0.40	-3.45	7.08	0.66
**S11**	1.90	12.90	13.66	30.98
**S12**	-0.89	-2.77	13.01	4.45
**S13**	-8.57	-13.10	-0.62	-21.64
**S14**	0.13	-0.78	0.00	-0.88
**S15**	0.00	0.00	0.00	0.00
**S16**	-1.53	-4.09	-3.92	-9.24
**S17**	-0.16	-0.77	1.40	-0.01
**S18**	-1.92	12.73	14.59	23.99
**S19**	-9.31	2.70	-4.38	-12.70
**S20**	1.11	-3.00	-1.88	-1.99
**S21**	-2.11	3.49	-14.60	-9.56
**S22**	8.50	7.38	-21.61	4.92
**S23**	-0.48	0.78	8.40	7.33
**S24**	1.28	-1.57	0.84	1.01
**S25**	0.00	15.34	-15.81	0.00
**S26**	14.08	-14.69	22.75	26.15
**S27**	69.93	21.28	3.30	148.98

#### 4.3.2. The between-group consumption substitution effect

The between-group consumption substitution effect is another important factor that can be used to analyze variation in household indirect energy consumption. Data presented in Tables 3 and 5 summarize decomposition results; these show that the between-group substitution effect increases sectoral indirect energy consumption in the non-energy-intensive sector by 30.57 Mtce (i.e., 6.98% compared with indirect sectoral energy consumption in 1997) and decreases indirect energy consumption by 3.43 Mtce (i.e., 4.64% compared to sectoral indirect energy consumption in 1997) between 1997 and 2012. It is clear that the between-group substitution effect for specific sectors contributes to decreasing household sectoral indirect energy consumption in nearly all energy-intensive sectors while also increasing consumption in all non-energy intensive areas. Although this effect is relatively small, there is nevertheless good evidence that the between-group consumption substitution effect helps to reduce household indirect energy consumption.

**Table 5:** Comparison of between-group consumption substitution effects: Contribution to household indirect sectoral energy consumption change (unit: Mtce).

Sector code	1997–2002	2002–2007	2007–2012	1997–2012
**S1**	-1.82	2.31	3.82	4.79
**S2**	-0.17	0.07	0.16	0.25
**S3**	-0.06	0.00	0.00	0.00
**S4**	0.00	0.00	0.00	0.00
**S5**	-0.02	0.00	0.00	0.00
**S6**	-2.08	5.05	5.44	7.11
**S7**	-0.36	0.16	0.26	0.32
**S8**	-1.02	2.16	1.11	1.30
**S9**	-0.20	0.21	0.17	0.23
**S10**	-0.26	0.20	0.28	0.35
**S11**	0.18	-0.99	-0.20	-0.15
**S12**	1.08	-1.64	-1.21	-1.71
**S13**	0.66	-0.25	-0.22	-0.29
**S14**	0.03	0.00	0.00	0.00
**S15**	0.00	0.00	0.00	0.00
**S16**	-0.36	0.30	0.10	0.14
**S17**	-0.06	0.04	0.06	0.08
**S18**	-0.38	1.32	0.58	0.77
**S19**	-0.66	1.12	0.88	1.17
**S20**	-0.67	0.78	0.55	0.72
**S21**	-0.31	0.64	0.08	0.13
**S22**	1.29	-1.85	-0.83	-1.28
**S23**	-0.30	0.33	-0.01	-0.12
**S24**	-0.24	0.23	0.07	0.07
**S25**	0.00	0.95	0.00	0.00
**S26**	-1.47	1.96	2.08	2.46
**S27**	-6.78	12.57	8.59	10.80

#### 4.3.3. Comparison of how consumption-related effects contribute to sectoral household indirect energy consumption changes

**Fig 4:**
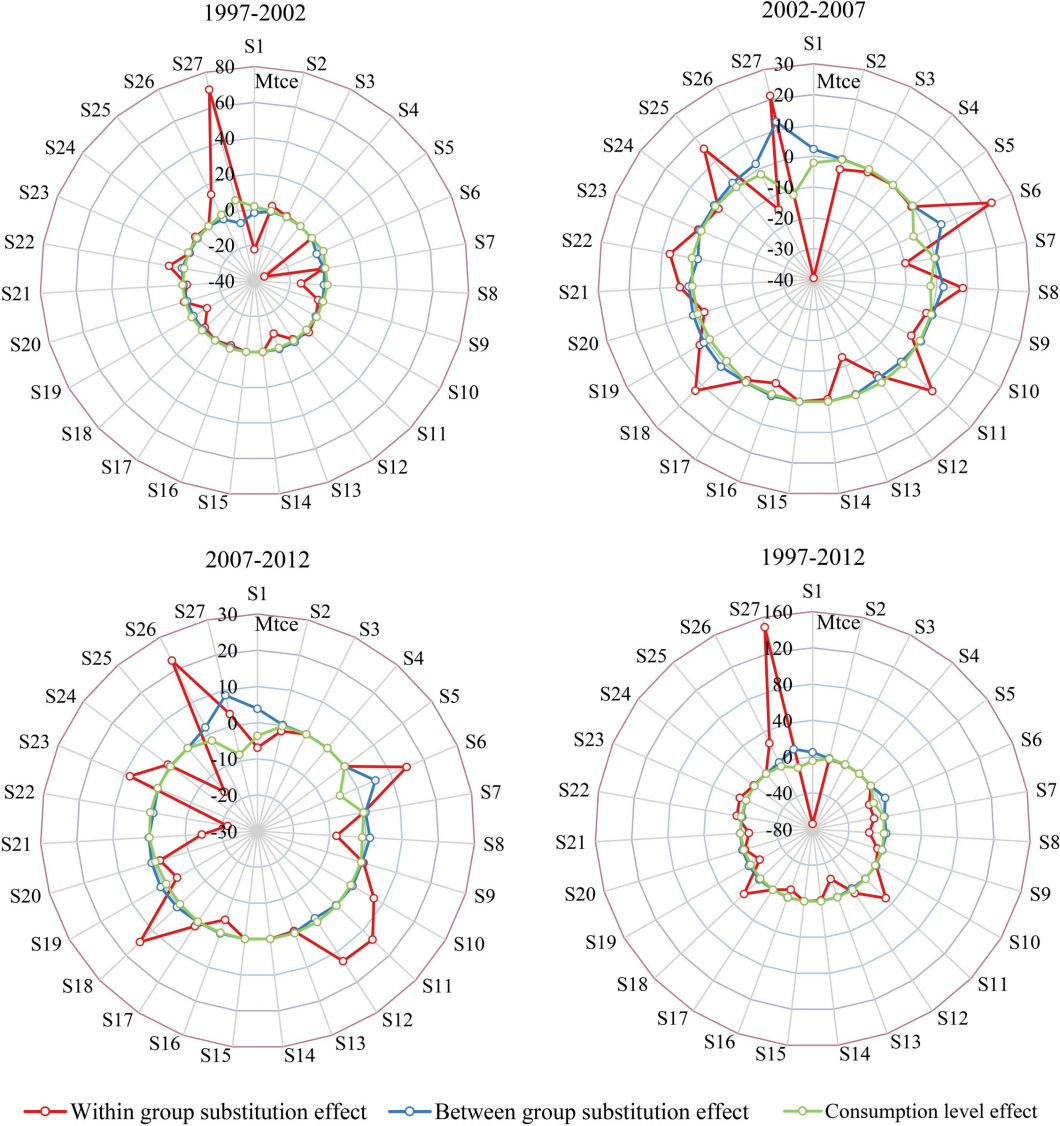
Comparison of consumption-related effect contributions to sectoral household indirect energy consumption changes between 1997 and 2002, 2002 and 2007, 2007 and 2012, and 1997 and 2012.

The data summarized in Fig 4 show that the within-group substitution effect contributes to the most significant changes in the bulk of sectoral household indirect energy consumption across all periods. In contrast, it is also clear that between-group substitution and consumption level effects contribute less to changes in household indirect energy consumption. The within-group substitution effect did not reveal obvious evidence that the consumption of high energy intensity sectors was substituted by others with low energy intensities over the time period of this analysis. It is nevertheless clear that the between-group effect reduced the household indirect energy consumption of energy-intensive sectors between 2002 and 2007, between 2007 and 2012, and between 1997 and 2012. Similarly, between 2002 and 2007 and 2007 and 2012, the between-group substitution effect exerted a very similar result on most sectors within these two periods while the within-group substitution effect had a relative divergent impact on some sectors.

## 5. Conclusions and policy implications

### 5.1. Conclusions

A two-tier structural decomposition method was used here to examine the relationship between consumption substitution and Chinese household indirect energy consumption between 1997 and 2012. Results show that household indirect energy consumption increased 161.2% between 1997 and 2012 and that indirect energy consumption accounted for between 75% and 78% of total household energy consumption over this period. First-tier decomposition results indicate that the energy intensity effect has contributed most to the decrease in household indirect energy consumption while the consumption scale effect contributed most to the concomitant increase. The household consumption structural effect was responsible for a slight rise over the time period of this analysis. Second-tier decomposition results show that the within-group substitution effect has increased sectoral household indirect energy consumption in both energy-intensive and non-energy intensive sectoral groups. There is also strong evidence that the between-group substitution effect had an impact on reducing household indirect energy consumption between 1997 and 2012.

### 5.2. Policy implications

Growth in consumption is an irresistible trend in China as this stimulates economic growth. Indeed, this trend is becoming an increasingly critical issue to the national economy. On the basis of the results presented in this article, a number of policy recommendations are proposed to meet growing energy consumption challenges in the face of ever-increasing consumption.

(1) Energy intensity is the most important factor to consider when reducing household indirect energy consumption. The Chinese government should therefore continue to support growth and investment in research and development, especially with regard to increasing energy efficiency and mitigating the intensity of energy-intensive sectors. The indirect energy consumption of energy-intensive products will decrease as a result.

(2) Considerable divergence in energy intensities are seen even within a single sectoral group. Thus, although the within-group consumption substitution effect is not currently prominent, this can happen more easily within a single sectoral group; the government should therefore utilize tax and subsidy policies to promote within-group consumption substitution. This can emphasize the increased consumption of relatively low energy-intensive products rather than alternatives with the same function.

(3) Results show that substitution between non-energy intensive and intensive sectors adds significantly to indirect consumption. Thus, from the perspective of saving consumption energy, government policy should stimulate the consumption of non-energy-intensive goods as substitutes for energy-intensive ones. As this will increase the cost of energy intensive products and stimulate consumption substitution between groups, higher taxes on more energy intensive products would be practical.

Mitigating the effects of growth in energy use is a long-term challenge in China. There remains much work to do to address prevailing attitudes and practices in production and consumption before this goal can be fully attained. The outcomes of this research show that consumption substitution can be effective in reducing household indirect energy use and that there is an enormous potential for this phenomenon to substantially reduce energy use in China. The question of how to promote consumption substitution to reduce energy use will comprise one important research area in the future.

## Supporting information

S1 Appendix (DOCX)
