# Correction: Consumption substitution and change of household indirect energy consumption in China between 1997 and 2012

**DOI:** 10.1371/journal.pone.0246780

**Published:** 2021-02-04

**Authors:** Zhipeng Tang, Shuang Wu, Jialing Zou

In the Second-tier decomposition of household consumption structure subsection of the Data and methods, a variable is missing from the equation in the second sentence of the fourth paragraph. Please view the complete, correct equation here:

$$\Delta z_{be}=r_{t_{0}\to t_{1}}^{EI}z_{jm(t_{1})}^{NEI}+r_{t_{0}\to t_{1}}^{NEI}z_{jm(t_{1})}^{EI}-r_{t_{0}\to t_{1}}^{EI}z_{jm(t_{1})}^{EI}-r_{t_{0}\to t_{1}}^{NEI}z_{jm(t_{1})}^{NEI}$$

The subheading 4.1.2. Household indirect energy consumption is incorrectly located in the caption for Fig 1. The subheading should be located above the second paragraph in the Results section. Please see the correct Fig 1 caption here.

**Fig 1:**
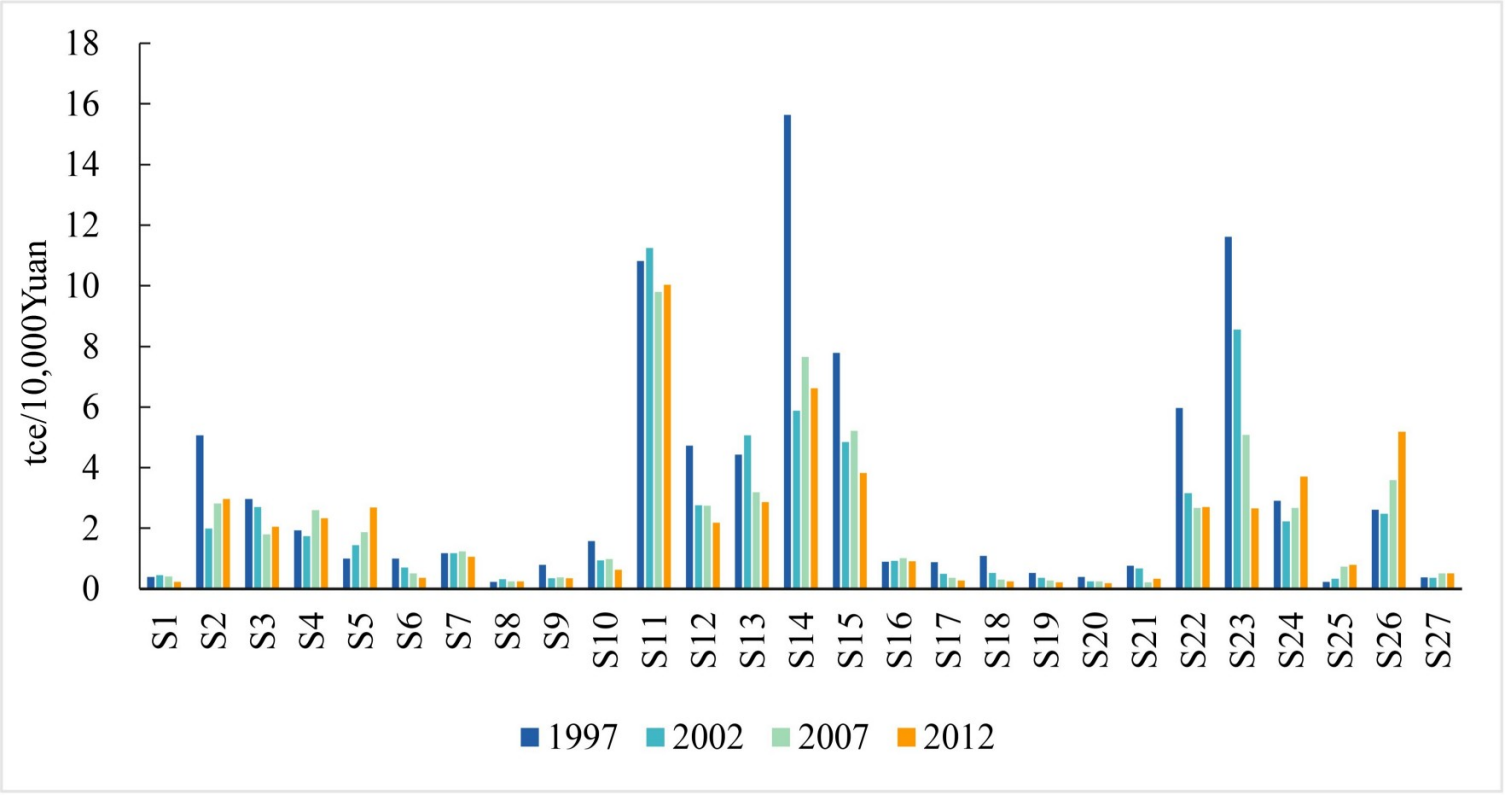
Comparison of Chinese sectoral energy intensities in 1997, 2002, 2007, and 2012.
