# Supplementary material for: Consumption substitution and change of household indirect energy consumption in China between 1997 and 2012
Source: PLoS One. 2020 Aug 28;15(8):e0221664. doi: 10.1371/journal.pone.0221664 (PMC7454985; doi:10.1371/journal.pone.0221664)
Supplement: S1 Appendix — (DOCX) [file pone.0221664.s001.docx]

Appendix A

Proof Eq. (10) and Eq. (11):

Eq. (6) is as follows:

| $s_{j}=\sum_{m} s_{jm}=\sum_{m} z_{jm}q_{m}$ | (A.1). |
| --- | --- |

It is possible to derive:

| $\frac{s_{j}^{t_{1}}}{s_{j}^{t_{0}}}=\frac{\sum_{m} s_{jm}^{t_{1}}}{\sum_{m} s_{jm}^{t_{0}}}=\frac{\sum_{m} z_{jm}^{t_{1}}q_{m}^{t_{1}}}{\sum_{m} z_{jm}^{t_{0}}q_{m}^{t_{0}}}$ | (A.2). |
| --- | --- |

This expression can be further expanded as follows:

| $\ln\left( \frac{s_{j}^{t_{1}}}{s_{j}^{t_{0}}} \right)=\sum_{m} \frac{\frac{s_{jm}^{t_{1}}-s_{jm}^{t_{0}}}{\ln\left( s_{jm}^{t_{1}} \right)-\ln\left( s_{jm}^{t_{0}} \right)}}{\frac{s_{j}^{t_{1}}-s_{j}^{t_{0}}}{\ln\left( s_{j}^{t_{1}} \right)-\ln\left( s_{j}^{t_{0}} \right)}}\cdot\ln\left( \frac{z_{jm}^{t_{1}}}{z_{jm}^{t_{0}}} \right)+\sum_{m} \frac{\frac{s_{jm}^{t_{1}}-s_{jm}^{t_{0}}}{\ln\left( s_{jm}^{t_{1}} \right)-\ln\left( s_{jm}^{t_{0}} \right)}}{\frac{s_{j}^{t_{1}}-s_{j}^{t_{0}}}{\ln\left( s_{j}^{t_{1}} \right)-\ln\left( s_{j}^{t_{0}} \right)}}\cdot\ln\left( \frac{q_{m}^{t_{1}}}{q_{m}^{t_{0}}} \right)$ | (A.3). |
| --- | --- |

Thus, Eq. (7) and the function of logarithmic mean Divisia weight value $L(x)$ can be expressed as follows:

| $\Delta s=\Delta z+\Delta q=\sum_{j} L\left( E_{j} \right)\sum_{m} \frac{L\left( s_{jm} \right)}{L\left( s_{j} \right)}\cdot\ln\left( \frac{z_{jm}^{t_{1}}}{z_{jm}^{t_{0}}} \right)+\sum_{j} L\left( E_{j} \right)\sum_{m} \frac{L\left( s_{jm} \right)}{L\left( s_{j} \right)}\cdot\ln\left( \frac{q_{m}^{t_{1}}}{q_{m}^{t_{0}}} \right)$ | (A.4). |
| --- | --- |
